# Bioadhesive Curcumin-Mediated Photodynamic Inactivation and Its Potential to Cause Undesirable Effects on Dental and Restorative Surfaces

**DOI:** 10.3390/pharmaceutics13091458

**Published:** 2021-09-13

**Authors:** Bárbara Donadon Reina, Carolina Santezi Neto, Patrícia Petromilli Nordi Sasso Garcia, Marlus Chorilli, Giovana Maria Fioramonti Calixto, Lívia Nordi Dovigo

**Affiliations:** 1 Department of Social Dentistry, School of Dentistry, São Paulo State University (UNESP), Rua Humaitá 1680, Araraquara SP 14801-903, Brazil; barbara.reina@unesp.br (B.D.R.); patricia.garcia@unesp.br (P.P.N.S.G.); 2 Independent Researcher, the Moment of the Submission (Unaffiliated Researcher), São Carlos SP 14801-903, Brazil; carolina_santezi@hotmail.com; 3 Department of Drugs and Medicines, School of Pharmacy, São Paulo State University (UNESP), Rodovia Araraquara-Jaú, Km. 1, Araraquara SP 14800-903, Brazil; marlus.chorilli@unesp.br; 4 Department of Biosciences, Piracicaba Dental School, University of Campinas (UNICAMP), Avenida Limeira, Piracicaba SP 13414-903, Brazil; gcalixto@unicamp.br

**Keywords:** liquid crystals, photochemotherapy, photosensitizing agents, curcumin, composite resins

## Abstract

Curcumin-mediated Photodynamic Inactivation (PDI) has shown great potential to disinfect specific sites on tooth enamel but may involve contact with restorative materials. Thus, before use in dentistry, it is necessary to investigate whether the PDI protocol causes undesirable changes in the surfaces of aesthetic restorative materials and dental enamel. This study investigated the effect of PDI mediated by curcumin (CUR) in a liquid crystal precursor system on color stability (ΔE), surface roughness (Ra), and microhardness (kgf) of three different composite resins and bovine dental enamel specimens. The microhardness and roughness readings were performed 60 days after the treatments while the color readings were performed immediately, 24, 48, and 72 h, 7, 14, 21, 30, and 60 days after the treatments. Results showed that CUR mediated-PDI does not seem to have the potential to promote any esthetic or mechanical changes to the surface of tooth enamel and can be applied safely in clinical practice. However, the results on color, roughness, and hardness obtained for composite resins show that some negative effects can be produced, depending on the type of restorative material; more experiments must be performed with different formulations and, perhaps, with lower concentrations of CUR.

## 1. Introduction

Caries is still the most common disease in the world with 3.5–5 billion people suffering from it [1], and remains the main reason for tooth loss by, mainly, the reduction of mechanical properties [2,3]. Furthermore, caries can promote systemic complications for the patient, along with its impacts on the index of disability-adjusted life-years and edentation, which has a major influence on quality of life [1]. Microorganisms are associated with the development of caries and other oral conditions, such as periodontal and endodontic diseases, oropharyngeal candidiasis, prosthetic stomatitis, bad breath, and peri-implantitis [2,4,5,6,7]. These microorganisms colonize dental and prosthetic structures through biofilm formation, the main factor associated with the development of oral infections [8]. In addition to their relationship with the onset of infections, biofilms may cause a decrease in susceptibility to antimicrobial agents, hindering drug action, and increasing resistance to different agents [9,10].

Despite the high prevalence, most oral diseases can be prevented or treated in their early stages [5]. Further, it is known that the lack of an overview is what causes the main failures of modern dentistry, generating defective dental care models. However, regardless of the level of dental treatment achieved, this will not end the problem of caries in the world [11,12]. Therefore, it is necessary that we seek new approaches to combat this global challenge, moving further away from an exclusively interventionist approach. Thus, caries will only be prevented and reversed with adequate resources [13,14]. This is also in line with a specialized and increasingly technological qualification, characteristic of Dentistry 4.0 in which we are inserted, but which must always be concerned with the safety of both the patient and the dentist.

One way to prevent oral diseases is to combat microbial biofilms and, consequently, associated infectious diseases, using locally acting antimicrobials. The increasing incidence of infectious diseases and the emergence of resistance of microorganisms to conventional antiseptics, antibiotics, and antifungals, have been directing research towards alternative antimicrobial treatments [15,16,17,18]. Photodynamic Inactivation (PDI) has been pointed to as a promising therapeutic modality for inactivation of pathogenic microorganisms [19,20,21,22]. The photodynamic process requires the use of a photosensitizing agent (FS), the application of a light that corresponds to the absorption band of the FS, and the presence of oxygen. Light-excited FS can react with neighboring molecules by transferring electrons or hydrogen (type I reaction) or by transferring energy to oxygen (type II reaction), leading to the production of reactive species. Both pathways may lead to cell death, and the occurrence of the type of reaction may be related to the characteristics of the FS employed. Reactive species formed during the photodynamic process have non-specific reactivity with organic molecules; thus, any cellular macromolecule can be a potential target [23,24].

Because it is a minimally invasive approach with great antimicrobial potential, PDI has been investigated in several dentistry fields, helping to remedy the deficiencies of currently available antimicrobial treatments. Still, PDI can be used for both the treatment and prevention of oral diseases, because in addition to treating the pathology, site-specific action results in the occurrence of adverse effects rarely being mentioned, and the development of microbial resistance is very unlikely (which makes it safer to use it as a prevention as well) [25]. Both oral cavity bacteria and fungi, including those involved with the carious process, are susceptible to different PDI protocols [26,27,28,29]. Specifically, PDI can be used as a method of disinfection or microbial control, as well as bacterial reduction in root canals, periodontal pockets, sites with peri-implantitis, oral decontamination before surgery, and caries lesions. Curcumin (CUR), a natural pigment extracted from the rhizomes of the *Curcuma longa* L. plant and known as turmeric, has high light absorption capacity at wavelengths close to blue and has been identified as a potential FS for elimination of microorganisms. However, CUR has water-solubility issues, so to make its clinical applications feasible, pharmacological alternatives are proposed for its delivery.

Liquid crystalline systems (LCS) are considered ordered structures with molecular arrangement characterized by alternating hydrophobic and hydrophilic regions, which appear to be an excellent lipophilic drug release matrix [30,31,32] that can optimize CUR efficiency. Furthermore, LCS can incorporate water present in saliva to promote structural changes and result in a more viscous and mucoadhesive liquid crystalline system in the oral cavity [33,34]. In order to facilitate the clinical application of the formulation, a precursor of liquid crystalline system (LCP), which is a liquid formulation, can be designed to become an in-situ viscous liquid crystalline system. Thus, the incorporation of CUR in a LCP previously developed by our research group [33], containing polyoxypropylene-(5)-polyoxyethylene-(20)-cetyl alcohol, oleic acid and C974P dispersion, can present advantages such as high viscosity and bioadhesion to the teeth after contact with saliva, besides presenting reduced toxicity.

In addition, before the use of PDI in dentistry it is necessary to investigate whether the proposed PDI strategy causes undesirable changes in the surfaces of aesthetic restorative materials and dental enamel. CUR, like other FS used in PDI, is a substance that may have coloring characteristics. Therefore, color may change on the surfaces exposed to this compound. Also, there is the possibility of changes in other surface properties of materials in contact with reactive species formed during PDI, such as hardness and roughness of composite resins and dental enamel. If topical application of PDI could promote color changes in the dental aesthetic restorations and tooth enamel, or even impair their surface properties such as hardness or surface roughness, it cannot be safely recommended. Increasing surface roughness could promote greater accumulation of dental plaque [35,36,37] while changes in hardness could decrease surface resistance to abrasion, fractures, decreased flexural strength and compressive strength, which would directly influence tooth health and longevity of esthetic restorations [38,39,40,41]. Therefore, this study evaluated whether PDI with curcumin, incorporated in LCP, can promote changes in surface roughness, microhardness, and color of resinous materials and dental enamel.

## 2. Materials and Methods

### 2.1. Experimental Design

This was a double-blind laboratory-based experimental study, in which the researcher responsible for the measurements and statistics was blinded. For the analysis of the effects on restorative materials, the response variables considered were color stability (ΔE), surface roughness (Ra; µm), and microhardness (Vickers test; kgf). The independent variables were the type of composite resin (a. TPH Spectrum Resin by Dentsply, b. Amelogen^®^ Plus by Ultradent; and c. Filtek™ Z350 Supreme Ultra Universal Restorative by 3M), the antimicrobial treatment (a. NC: negative control—only artificial saliva; b. LCP: positive control; a liquid crystal precursor system without curcumin; c. PDI: photodynamic inactivation), and time of evaluation (color stability under 8 conditions: 24 h, 48 h, 72 h, 7 days, 14 days, 21 days, 30 days, and 60 days after treatment). Immersion in artificial saliva in the first 24 h was treated as the baseline. For the analyses of the effect on the surface of bovine tooth enamel, the response variables considered were color stability (ΔE), surface roughness (Ra; µm), and microhardness (Knoop hardness test; kgf). The independent variables were the same treatments and evaluation times used in the previous analysis.

The data on color, roughness, and microhardness was collected by a trained and calibrated researcher (ρ ≥ 0.894). The sample size was calculated using the GPower software and with α = 0.05 and β = 0.20. The minimum sample size for the measurements was 20 samples per group for the three types of composite resin and 30 samples per group for the enamel specimens.

### 2.2. Preparation of the Precursor of Liquid Crystalline System

The precursor of liquid crystalline system was composed of 20% aqueous dispersion of Carbopol 974P (C974P, Lubrizol, Cleveland, OH, USA) at 0.5% as the aqueous phase, 40% oleic acid (Synth^®^, Diadema, São Paulo, Brazil) as the oil phase, and 40% ethoxylated 20 OE and 5% propoxylated alcohol surfactant (PPG-5-CETETH-20, Procetyl^®^ AWS, Croda, Campinas, São Paulo, Brazil). For its preparation, 10% of 5.0% C974P (*m*/*m*) was mixed with 10% high purity water in order to produce a final polymeric percentage of 0.5% in the LCP. Then, it was slowly poured and mixed with the 40% PPG-5-CETETH-20 and 40% oleic acid mixture for 2 min by magnetic stirring (Fisatom^®^, Sao Paulo, SP, Brazil) at 300 rpm. The incorporation of CUR into the LCP (CUR-LCP) was performed by adding 80 µM of CUR (79%, Sigma-Aldrich, San Louis, MO, USA) to the oleic acid [33,42].

### 2.3. Materials Used to Create the Test Specimens

Three different composite resins were used in the color A2 (Table 1). In addition, the tooth enamel specimens were created using the coronal portion of bovine teeth (authorized by the local animal ethics committee [Ethics Committee on the use of animals at the Araraquara School of Dentistry—CEUA FOAr] 13/2015, 14 August 2015).

### 2.4. Creation of the Test Specimens

**Figure 1:**
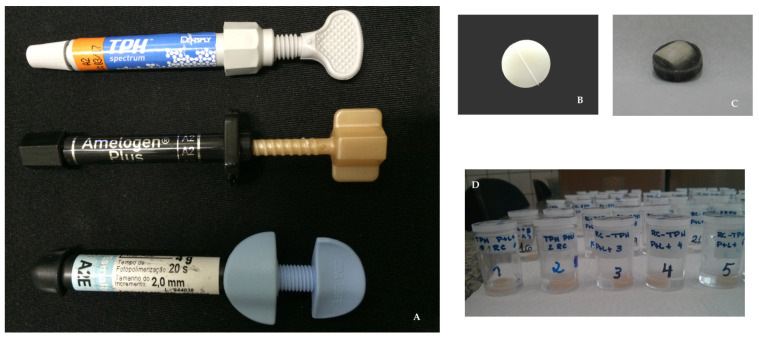
The composite resins used (**A**), resin (**B**) and bovine enamel (**C**) specimens, and the specimens randomization procedure (**D)**.

The composite resin test specimens (Figure 1B) were created following the methodology described in the literature [43], which resulted in one groove in the inferior portion of the test specimen. This marking is necessary to distinguish between the polished and unpolished sides, as well as to correctly position the test specimen to perform the microhardness, roughness, and color readings so that the data can be consistently collected with the specimens in the same position. After the fabrication and polishing processes, the test specimens were separated by the composite resin used and stored in properly identified and moisture-free dark-colored containers until randomization.

The test specimens created in tooth enamel relied on bovine teeth (Figure 1C) that were first kept under running water for 5 h and then stored in 0.2% thymol (15 days; 5 °C). After this period, the teeth were scraped and smoothed from the coronal portion to the root. The organic content of the root pulp was aspirated, and the teeth were placed in a new 0.2% thymol solution (15 days; 5 °C). After that, the teeth were washed in running water (5 h) and immersed in a container with distilled water (24 h) in order to remove thymol residues. The teeth were then polished using pumice powder. The teeth were sectioned after fixation using impression material heated in a wooden mold, and the mold with the teeth inside was positioned in the slicing machine (IsoMet 1000, Buehler, Lake Bluff, IL, USA). The first cut was made at a speed of 250 G (position 0 mm to separate the root portion from the coronal portion. Next, using the same disk rotation speed and without removing the previously sectioned root portion, the wooden mold was repositioned to 7.3 mm, and the second cut was made to produce a third section that included the coronal portion of the tooth. The cervical third of the dental crown was discarded, and the portion representing the middle third (7 mm × 7 mm) was used. Because the machine used to make the sections does not allow for a consistent desired thickness (2 mm) to be established, once the enamel blocks were fabricated, they were worn down using a rotary grinder drill.

After the enamel blocks were made, they were kept under running water for 5 h, placed in acrylic resin (color 67 pigmented with Pó Xadrez^®^ compact pigment; LanXess; Bayer) to turn them a light gray color with the aid of a circular silicone matrix. After the placement of the test specimen in acrylic resin, a disc (10 mm in diameter and approximately 3 mm in thickness) was obtained. It was then polished using a 400-grit water file). Its surface contained the vestibular surface of the enamel block, and its backside was smoothed using the water file at increasing grit levels (400, 600, and 1200) positioned in a DP 10 polisher (Panambra Struers DP—10, Panambra, São Paulo, Brazil) to acquire the desired thickness of 2 mm.

### 2.5. Measurement Procedures

Twenty-four hours before the baseline readings, the specimens of each material were randomly divided and separated into individual plastic containers (Figure 1D) according to the surface type and the group to which they belonged (NC, LCP, and PDI). Next, 1000 µL of artificial saliva was added to each plastic container. The composition of the artificial saliva was as follows (g/500 mL): glucose, 0.015; NaCl, 0.290 g; CaCl_2_, 0.085 g; Na_2_HPO_4_, 0.17 g; NH_4_Cl, 0.08 g; KCl, 0.635 g; NaSCN, 0.080 g; KH_2_PO_4_, 0.165 g; urea, 0.1 g; double distilled water, 500 mL [44]. The containers were then placed in the microbiological incubator at 37 °C for 24 h. The containers were numbered in ascending order (1 to 180 for the test specimens in composite resin and 1 to 90 for the bovine enamel test specimens) so that the researcher responsible for the evaluations remained blind to the groups.

**Figure 2:**
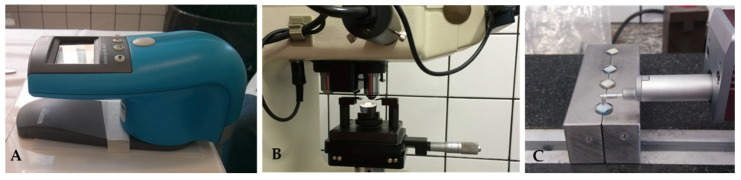
The measurement procedures used: color reading by a colorimetric spectrophotometer (**A**); microhardness, using a digital microhardness tester machine (**B**); and the surface roughness, measured by a surface roughness measuring instrument (**C**).

The order in which the baseline reading procedures were performed was color reading, roughness reading, and then microhardness reading. A colorimetric spectrophotometer (Color guide 45/0, PCB 6807 BYK-Gardner GmbH, Gerestsried, Germany) (Figure 2A) was used to measure color between wavelengths of 400 and 700 nm using direct transmission, with standard D65 illumination on a white background plus an auxiliary apparatus to measure the color. For the color readings, the test specimens were removed one by one from their containers, and the excess moisture was removed using gauze. After each reading, the specimens were returned to their respective acrylic containers. All of the values were recorded and calculated by the device. After the color of each sample was assessed in the different periods, the ΔE (based on the ΔL*, Δa*, and Δb* values using the CieLab technique) were calculated to analyze the differences between the baseline values and subsequent values.

The color readings were immediately followed by the surface roughness readings (Ra; µm). The readings were obtained by passing the diamond tip (5 μm radius) of the portable surface roughness measuring instrument (Surftest SJ-401, Mitutoyo Corporation, Kawasaki, Japan) (Figure 2C) over the test specimen (2.5 mm in length) at a speed of 1 mm/s with an accuracy of 0.01 μm. This procedure was performed in three different spots, the measurements of which were averaged to obtain a final mean Ra for each test specimen. A matrix (like the one used to prepare the specimens in resin) was used to standardize the readings. The matrix was two-sided and had a central marking to guide the consistent positioning of the specimens in the same direction, one which coincided with the groove on the backside of the test specimen with the two-sided matrix marking. Parallel to this central line, two other lines (one 2 mm above the central line and one 2 mm below the central line) were marked on the matrix. Another line was drawn perpendicularly to the lines and created an imaginary line down the middle of each test specimen. The intersection of these lines formed three points that guided the positioning of the diamond tip of the Surftest to standardize the reading.

These steps were followed by the microhardness measurements according to the Vickers and Knoop tests. The measurements were obtained using a digital microhardness tester machine (Buehler-Lake Bluff, IL, USA) (Figure 2B). A load of 50 kgf was applied to the composite resin test specimens for 30 s, and the reading was performed at three distinct points, which were then averaged to produce a final mean value for each specimen. To standardize readings and guide correct positioning, the test samples were placed in a matrix so that the groove behind the specimen coincided with the matrix groove. The coordinate used to guide the indentation of the center point was 13 right/left, with coordinate 10 guiding the anterior position, and coordinate 16 guiding the position posterior to the central indentation. For the bovine tooth enamel specimens, the coordinates used to guide the 3 indentation points were 5, 4, and 6, and the load used was 50 kgf for 15 s.

At the end of the readings, the plastic containers that held the test specimens along with 1000 μL of artificial saliva were returned to the incubator for 24 h at 37 °C, at which point the treatments were performed.

### 2.6. Application of the PDI Protocol to the Test Specimens

In this study, CUR (79%, Sigma-Aldrich) was used in a LCP system (LCP-CUR), the antimicrobial efficacy of which has been evaluated in a previous study [45]. The final CUR concentration in the LCP-CUR was 80 µm. This formulation was used in the PDI group along with proper illumination. The light source was a device with light emitting diodes (LED) that emitted light predominantly at 455 nm, which allowed for the uniform and simultaneous illumination of all of the wells of each plate with 22 mW/cm^2^ of intensity. The amount of light evaluated was 18 J/cm^2^ (14 min). In the positive control group, the same LCP was used, but without the addition of CUR (LCP group). The specimens belonging to the negative control (NC) groups did not undergo any treatments.

The plastic containers in which the test specimens were immersed in artificial saliva were divided by group (NC, LCP, and PDI), specimens were washed with distilled water and transferred individually into the wells of the 24-well plates. For the PDI treatment, 500 μL of LCP-CUR was pipetted into the wells of the 24-well plate, and the specimens were immersed in the formulation for 5 min in the dark (pre-illumination time, or PIT). The plate was then placed in the light device for 14 min. The positive control group (LCP) was used to determine whether the liquid crystal precursor system alone could change the surfaces tested. To this end, 500 μL of the CUR-free system was pipetted into the wells of the plate and the specimens in this group were immersed in the formulation for 19 min in a dark environment (5 min of which were PIT and 14 min of which represented the illumination time of the PDI group). Specimens from the negative control (NC) groups remained in their respective plastic containers with artificial saliva inside an incubator at 37 °C until the color, microhardness, and roughness readings were performed.

After the treatments for both the LCP group and the PDI group, the specimens were removed from the wells, washed with distilled water to remove the formulation, and replaced in their respective containers with 1000 μL of artificial saliva. The test specimens were kept at 37 °C in an incubator and remained under these conditions until the readings were performed. The artificial saliva was changed weekly under aseptic conditions; at each change, the specimens were washed in distilled water to remove saliva deposits.

The readings performed after the treatments consisted of exactly the same methodology reported previously. The microhardness and roughness readings were performed 60 days after the treatments, while the color readings were performed immediately (referred to as 0 h), 24 h, 48 h, 71 h, 7 days, 14 days, 21 days, 30 days, and 60 days after the treatments.

### 2.7. Statistical Evaluation

The experimental design comprised three independent response variables (color, microhardness, and roughness) and data from each one of them were analyzed separately as follows. After checking for outliers, as well as normality and sphericity assumptions verification, a mixed repeated-measures analysis of variance (rANOVA) was performed. rANOVA with data from composite resins considered 2 independent factors (treatment and composite resin) and 1 paired factor (time). rANOVA with data from dental enamel considered 1 independent factor (treatment) and 1 paired factor (time). The Šidák correction (for color change and microhardness) and the Games–Howell post-hoc tests (surface roughness variable) were applied to resolve the issue of multiple comparisons (α = 0.05).

## 3. Results

### 3.1. Composite Resins

#### 3.1.1. Color Stability

**Figure 3:**
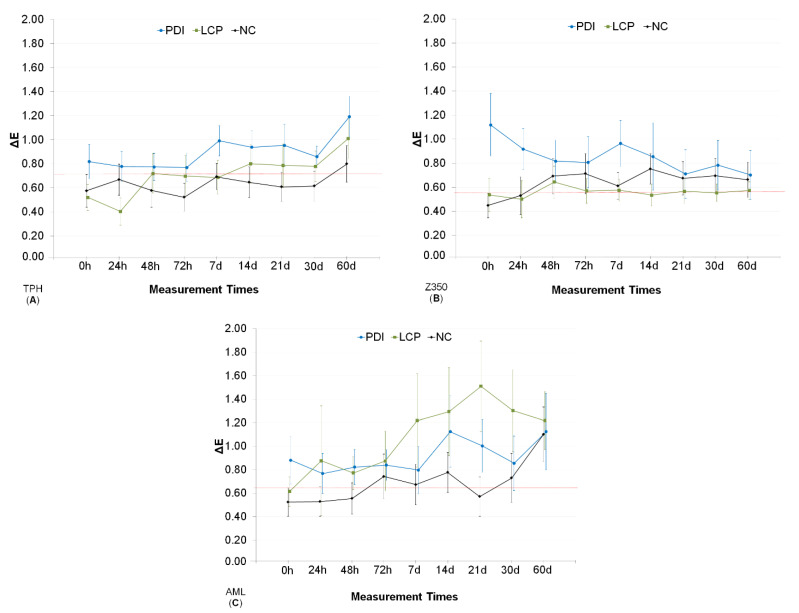
Mean and confidence intervals (CI 95%) for color changes (ΔE) obtained following treatment (PDI: curcumin-mediated photodynamic inactivation in a liquid crystal precursor system; LCP: only the liquid crystal precursor system without curcumin; NC: negative control with no treatment; only artificial saliva) at the different measurement times (0 h to 60 days). Type of composite resin: (**A**) is for TPH; (**B**) is for Z350; and (**C**) is for AML for comparison, the dotted red line indicates the upper limit of CI 95% from NC group at 0 h.

The results obtained at all of the color evaluation times can be seen in Figure 3. For TPH and Z350 resins, the mean values of color change in the PDI group were always higher compared to the control groups, although in many time points there was a crossing of the CIs limits, indicating the absence of statistical significance of the differences. Variability resulted in broad CIs limits, which is more noticeable for the AML resin. Despite this, color changes of less than 2 points were seen considering the broader upper limits, compared to the NC group. For all resins, data collected 60 days after treatments seem to indicate that PDI does not cause greater color change than would be naturally expected over time. In the mixed repeated measures ANOVA, only two time periods were considered (immediately after and 60 days after PDI). ANOVA results demonstrated that all the factors (time, treatment, and resin) had a significant effect on the change in color exhibited by the resins, as well as all possible interactions (*p* ≤ 0.037).

However, time was the factor that had the greatest effect size on the response variable (ɳ^2^_p_ = 0.204). The multiple comparisons analysis (Table 2) considered the interactions between the factors and revealed that the change in color caused by PDI for the TPH resin was not significant compared to LCP and CN groups. For the Z350 resin, the PDI group exhibited change in color when the period immediately after the treatments was considered. After 60 days, the change in color regressed at values statistically similar to the NC and LCP groups. Immediately after the treatments, the AML resin exhibited a change in color with PDI relative to the NC and LCP groups. This difference was not observed 60 days after treatment, at which time the color change values were found to be equivalent between the treatments. Thus, after 60 days, the color change caused by PDI was equivalent to the natural changes produced by saliva for all three resins.

#### 3.1.2. Surface Microhardness Analysis

The ANOVA results (Table 3) revealed that all of the factors were significant (*p* < 0.001) except for the “time vs. treatment” (*p* = 0.958) and “treatment” (*p* = 0.696) factors. The results from multiple comparisons are provided in Table 3. PDI was found to affect only the Z350 resin, which exhibited a reduction in hardness after the application of the treatment. For this resin, the application of the empty formulation did not result in a loss of hardness, confirming that the effect was due to the photodynamic action of curcumin. For the TPH and AML resins, both PDI and the application of the LCP did not change the hardness in comparison to the NC group. Overall, the AML resin exhibited lower hardness values than the other resins regardless of the treatment considered, with most of the values concentrated between 53 and 59 kgf.

#### 3.1.3. Surface Roughness Analysis

The analysis revealed that the effect of the “time” factor was not significant on roughness (*p* = 0.991), but the type of resin (*p* < 0.001), treatment (*p* < 0.001) and interaction “resin × treatment” (*p* < 0.001) had a significant effect. The results from multiple comparisons (Table 4) showed that the roughness of the TPH and Z350 resins did not change in the different groups, a finding which indicates that neither PDI nor LCP produced undesired changes to this variable. For the AML resin, there was a significant increase in roughness when the PDI group was compared to the NC group for both evaluation times. The empty formulation (LCP) also enhanced the roughness, with means values similar to the PDI, suggesting that the change may have been caused by the formulation itself, and not necessarily by the PDI. Additionally, the means of the NC groups for the TPH and Z350 resins did not differ significantly, suggesting that these resins have similar roughness. However, the roughness values of the NC group for the AML resin were lower than the others.

### 3.2. Bovine Tooth Enamel

#### 3.2.1. Color Stability

**Figure 4:**
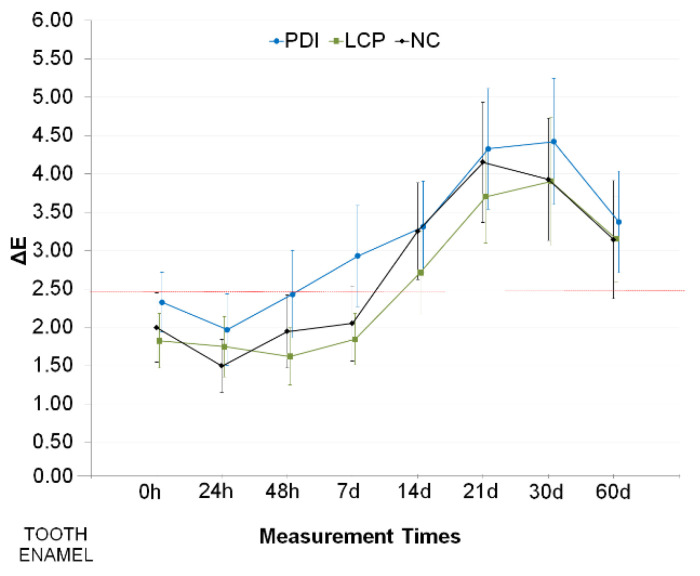
Mean and confidence intervals (CI 95%) for changes in color (ΔE) of bovine tooth enamel test specimens obtained following treatment (PDI: curcumin-mediated photodynamic inactivation in a liquid crystal precursor system; LCP: only the liquid crystal precursor system without curcumin; NC: negative control with no treatment; only artificial saliva), at the different measurement times (0 h to 60 days). For comparison, the dotted red line indicates the upper limit of CI 95% from NC group at 0 h.

**Figure 5:**
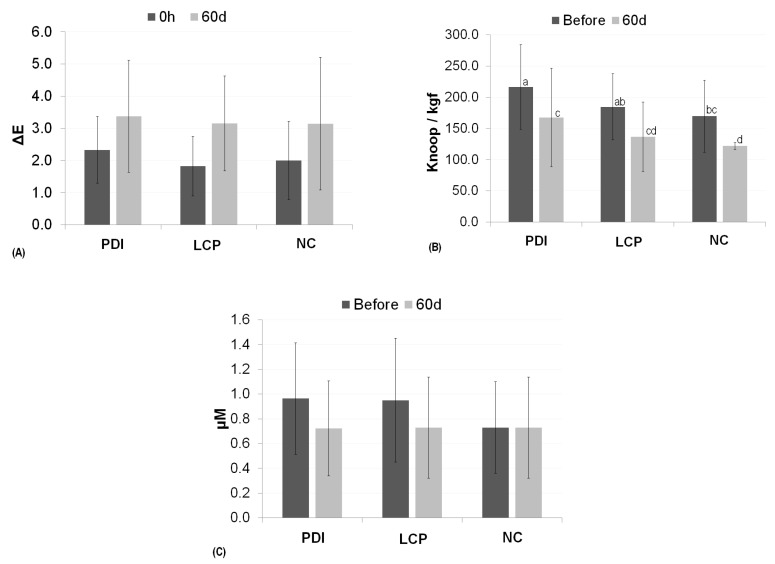
Mean and standard deviation for color changes (**A**), surface microhardness (**B**), and surface roughness (µM) (**C**), obtained on tooth enamel surfaces following treatment (PDI: curcumin-mediated photodynamic inactivation in a liquid crystal precursor system; LCP: only the liquid crystal precursor system without curcumin; NC: negative control with no treatment; only artificial saliva) at the different evaluation times. ‘0 h’ indicates color evaluations performed immediately after treatments; ‘Before’ indicates evaluations performed before treatments, and ‘60 days’ indicates evaluations performed after 60 days of treatment (stored in artificial saliva). In the chart labeled (**A**): Mixed ANOVA: time (*p* < 0.001); treatment (*p* = 0.444); time × treatment (*p* = 0.852). In the chart labeled (**B**): Mixed ANOVA: time (*p* < 0.001); treatment (*p* < 0.001); time × treatment (*p* = 0.996). Šidák correction (*p* correctio Columns marked with different lowercase letters (a, c, ab, cd, bc, and d) indicate a statistically significant difference among means according to Sidák correction. In the chart labeled (**C**): Mixed ANOVA: time (*p* = 0.002); treatment (*p* = 0.364); time × treatment (*p* = 0.080).

The ΔE values obtained in the eight evaluation periods suggest that the color change was time-dependent, mainly up to 21 days of follow-up (Figure 4). Mean values and its CIs indicated that color changes were quite similar among the three treatment groups. According to the ANOVA results, only the “time” factor contributed significantly to the change in enamel color (*p* < 0.001), while treatment (*p* = 0.444) did not. Regardless of the treatment, the measurements taken 60 days after PDI showed an increase in mean values of color change. (Figure 5A). These results suggest that the change in color occurred naturally over the evaluated period in which the test specimens were stored in artificial saliva.

#### 3.2.2. Surface Microhardness Analysis

Enamel hardness values before the treatments were significantly higher than the values obtained 60 days after treatment, regardless of the treatment performed (Figure 5B). These results indicate there was a reduction of hardness over time. In addition, difference in the hardness values between means pairs of PDI × LCP, and between LCP × NC groups were non-significant, regardless of the period at which the measurement was taken. Thus, surface hardness decreased in all of the groups during the period in which the test specimens were stored in artificial saliva, a finding which suggests that PDI did not trigger any negative effects on bovine tooth enamel surfaces.

#### 3.2.3. Surface Roughness Analysis

In the study of the surface roughness of the enamel surface, only the “time” factor was found to have a significant effect. In general, mean roughness before the treatments was significantly higher than the measurements performed 60 days after the treatments. This change suggests that PDI had no negative effects on the roughness of the enamel, and that the decrease observed was due to the natural behavior of this type of surface.

## 4. Discussion

Composite resins are restorative materials used in dental restorations after the removal of carious tissue. The resins largely consist of an organic matrix that surrounds an inorganic matrix, the former of which is composed of different types of resinous monomers such as bisphenol-A glycidyl methacrylate (BisGMA), urethane dimethacrylate (UDMA), or triethylene glycol dimethacrylate (TEDGMA). The inorganic matrix is formed by filler particles such as quartz, silica, barium, and strontium [37]. Depending on the size of the filler particle used, the resin will be classified as macroparticulate (particles ranging in size from 15 to 100 μm), microparticulate (particles of size 0.01 to 0.06 μm), hybrid (particles between 0.6 and 3.0 µm), microhybrid (particles between 0.4 and 2.0 μm), nanoparticulate (particles between 5 and 70 nm), or nanohybrid (particles between 0.04 and 3.0 μm) [46,47]. The particles dispersed in the organic matrix are responsible for giving the characteristics of the resins such as resistance to staining, higher or lower hardness, and more or less surface roughness [43].

This study investigated the possible effects of PDI on resin-based restorative materials. Because the finishing and polishing steps were consistent for all specimens, the changes in color observed here were caused by either the characteristics of the material evaluated, or the treatments performed [43]. The results for the TPH microhybrid resin showed that PDI did not produce any negative effects on the surface of the resin. A low amount of staining was also observed in studies that evaluated the color stability of microhybrid resins after immersion in dye solutions such as coffee, cola, and tea [48]. The compounds present in the LCP have a low affinity with the organic matrix of the resin, since the material did not dissolve and, as a consequence, did not contribute to the formation of gaps that could have trapped the dye molecules between the filler particles. The test specimens made with AML microhybrid resin were also found to exhibit no capacity for PDI staining. However, substantial variability was observed in all of the AML resin groups; for this reason, these results should be considered preliminary. Because both the TPH and AML resins were microhybrid, it was expected that the same behavior presented by the TPH resin specimens would be found in those made in AML, and there are, in fact, reports of similar behaviors for both of the microhybrid resins evaluated [35,37,43,48,49].

The only composite resin that presented a higher ΔE in the period immediately after PDI relative to its respective NC was the Z350 resin; however, 60 days after treatment, the ΔE decreased, meaning that the change in color found remained statistically equal to its NC. Because dyes can stain the resin through both absorption and adsorption [48,50,51], this result suggests that the change in color occurred superficially. It also suggests that the CUR residues may have been released from the surface of test specimens during the period in which the PDI group specimens remained in artificial saliva, which indicates that the staining is not permanent [35]. This finding for the Z350 resin has also been found by other authors who evaluated color stability [48,50], a consistency which suggests that Z350 resin is more susceptible to immediate resin. The resistance to changes in color that TPH seems to present relative to Z350 is associated with the composition, distribution, and dispersion of the filler particles in the organic matrix, a makeup which allows for a larger area of the organic matrix to be exposed to the dyes [36,48,49,50,51].

Wide confidence intervals were seen in color results and may indicate a certain inaccuracy of the measurements, which results in a lack of statistical significance given to the differences between means (a large amplitude of the intervals ends up facilitating the crossing of their limits). However, hypothesis tests confirmed the results that many differences could not be considered statistically significant. Furthermore, even in CIs with wider limits, it was possible to verify that the expected color change by PDI would not exceed 2 points. This data extends the practical interpretation of the color results because changes in ΔE of up to 3.3 is considered a clinical parameter of non-perceived color change to the naked eye [52].

The surface hardness of a given composite resin is closely related to its organic matrix and the filler particles present in its composition, as well as to the immersion media and polishing method used [43]. Thus, the lower resistance to the penetration of the diamond exhibited by the resin during the microhardness tests could be an indirect indicator of less resistance to wear from the chewing to which it would be exposed in the oral cavity. We observed that PDI produced no negative effects on TPH and AML resins, since, in all of the groups evaluated, hardness values did not differ significantly from baseline values when measured 60 days after treatment. For the Z350 resin, however, the specimens in the PDI group exhibited a significant reduction in hardness relative to the control when measured 60 days after treatment, a finding which suggests that CUR-mediated PDI harmed this property.

The resin hardness after exposure to bleaching agents was similar in the control and experimental groups of Z350 resin [36,53,54,55]; however, free radicals produced during the bleaching procedure can negatively affect the organic matrix-filler particle interface, contributing to the formation of gaps by rupturing the chemical bond between them, thereby reducing the hardness of the material [55]. Because the application of PDI also aids in the formation of free radicals, it can be suggested that the free radicals formed behave in the same way as those formed during dental bleaching procedures. Because of the wide variety of protocols used for bleaching, it is difficult to compare the effects that the bleaching procedure has on the hardness of resin-based materials to those produced by PDI. However, because the longevity of the resin restorations is directly dependent on esthetic, physical, and biological properties of the resins [36,53,54,55], the reduction in the hardness values observed in the test specimens in the PDI group for the Z350 resin may be considered a limiting factor for the use of PDI on oral cavity sites that are close to teeth restored with Z350. Hence, the roughness values of the TPH and Z350 resins were not altered in any of the test groups, results which indicate that neither PDI nor LCP made any significant changes to surface roughness. In addition, although the TPH and Z350 resins differ in their compositions, their surface roughness values were found to be similar. This result has also been reported by researchers who evaluated the surface roughness of microhybrid and nanoparticulate resins, and who found that, because of the characteristics of the filler particles in the Z350, its mechanical properties would have been as satisfactory as those found in hybrid and microhybrid resins [51,53,54]. In contrast, the specimens made using AML resin and in the PDI and LCP groups exhibited surface roughness values that were significantly similar to each other and increased when compared to those in the NC group. Therefore, the effects found herein may have occurred because of components in the LCP system, which could have altered the dispersion of the particles in the resin matrix, leading to a significant increase in surface roughness [53]. More studies are needed to analyze this behavior better.

In contrast to the results found for resin test specimens, those bovine tooth enamel specimens exhibited no changes that would suggest a possible influence of PDI on any changes in color, but rather a natural influence of artificial saliva over time. This conclusion can be made because, at the end of the 60-day study period, the changes in color were consistent across all groups. These results, therefore, suggest that these changes in color would have occurred naturally, regardless of the treatments performed. Meanwhile, studies that have evaluated the color stability of test specimens in tooth enamel after immersion in dye solutions have reported statistically significant changes in color relative to controls, findings which indicate that dyes are, in fact, a determining factor in variations in color [38]. Similar to that which was observed in the experiments on changes in color among bovine tooth enamel specimens, the “treatment” factor was found to not influence roughness and the “time” factor was responsible for the reduction in these values, as determined by the measurements performed 60 days after the treatments. The results found for the bovine tooth enamel test specimens differ from those obtained in studies that have evaluated the mechanical properties of enamel after dental bleaching, in which a reduction in microhardness has been observed; in the literature, microhardness was restored after at least 1 week of immersion in saliva artificial [41,44,56].

Therefore, CUR mediated-PDI does not seem to have the potential to promote any esthetic or mechanical changes to the surface of tooth enamel and can be applied safely in clinical practice. However, the results on color, roughness, and hardness obtained for composite resins show that some negative effects can be produced, depending on the type of restorative material, and more experiments must be performed with different formulations and, perhaps, with lower concentrations of CUR for the findings to be confirmed or refuted. A possible limitation of our study was that PDI was applied just once on the test surfaces; thus, further investigations need to evaluate the effect of repeated exposures to PDI protocols. The use of PDI to disinfect specific sites on tooth enamel may therefore be safer than general disinfection procedures, which may involve contact with restorative materials.

## Figures and Tables

**Table 1:** Compositions of the composite resins used in this study and their respective technical profiles.

Resin	Composition			Manufacturer
	Organic Content	Filler Particles	Other Components	
Amelogen^®^Plus (AML)	Bis-GMA	Non-specified particle measuring 0.7 µm in size	Not specified	Ultradent Products, Inc. South Jordan, UT, USA
Spectrum TPH resin (TPH)	Urethane-modified Bis-GMA	Silanized barium glass, aluminum borosilicate and silanized pyrolytic silica. Average size: 1.30 µm	Camphorquinone as a photoinitiator, EDAB, butylated hydroxytoluene, mineral dyes	DENTSPLY Ind. E Com. Ltd.a—Petrópolis, Rio de Janeiro, Brazil
Filtek™ Z350 Supreme Ultra Universal (Z350)	Bis-GMA, UDMA, TEGDMA, and Bis-EMA	Silica, zirconia, and zirconia/silica clusters	Not specified	3M EPSE Dental Products, St. Paul, MN, USA

**Table 2:** Mean values (±standard deviation) for the changes in color (ΔE) obtained after treatment (PDI: photodynamic therapy; LCP: Liquid crystal precursor without curcumin; NC: negative control/artificial saliva) and the type of composite resin (TPH, Z350, AML). ‘Immediate’ indicates evaluations performed immediately after treatments, and ‘60 days’ indicates evaluations performed after 60 days of treatment (stored in artificial saliva).

Composite Resin	Treatment	Evaluation Time	
		Immediate	60 Days
TPH	PDI	0.82 ± 0.31^Aa^	1.19 ± 0.37^CDa^
	LCP	0.52 ± 0.24^Aa^	1.01 ± 0.37^BCDb^
	NC	0.58 ± 0.30^Aa^	0.80 ± 0.33^ABCDa^
Z350	PDI	1.12 ± 0.58^Ba^	0.70 ± 0.45^ABCa^
	LCP	0.54 ± 0.30^Aa^	0.57 ± 0.16^Aa^
	NC	0.45 ± 0.22^Aa^	0.66 ± 0.32^ABa^
AML	PDI	0.88 ± 0.45^Aa^	1.12 ± 0.73^BCDb^
	LCP	0.61 ± 0.27^Ba^	1.22 ± 0.54^Da^
	NC	0.52 ± 0.26^Ba^	1.10 ± 0.51^BCDa^

The use of different letters next to the ‘mean ± sd’ values indicate a statistically significant difference among means: uppercase for comparison in the same column and lowercase for comparison in the same line. Mixed ANOVA: time (*p* < 0.001); treatment (*p* < 0.001); resin (*p* < 0.001); time × treatment (*p* = 0.008); time × resin (*p* < 0.001); resin × treatment (*p* = 0.448); time × resin × treatment (*p* = 0.037). Šidák correction (*p* ≤ 0.049).

**Table 3:** Mean values (±standard deviation) of hardness values as per the Vickers test (in kgf) obtained before treatment and after treatment (PDI: photodynamic therapy; LCP: Liquid crystal precursor without curcumin; NC: negative control/artificial saliva) and organized by composite resin (TPH, Z350, and AML).

Composite Resin	Treatment	Evaluation Time	
		Before	After
TPH	PDI	76.37 ± 5.82^Aa^	75.00 ± 9.12^Aa^
	LCP	75.97 ± 4.70^Aa^	67.02 ± 8.45^Aba^
	NC	76.91 ± 7.73^Aa^	69.88 ± 6.74^Aa^
Z350	PDI	85.17 ± 7.36^Aa^	60.67 ± 18.34^Bb^
	LCP	83.52 ± 4.36^Aa^	76.12 ± 7.34^Aa^
	NC	83.36 ± 4.50^Aa^	74.81 ± 8.18^Aa^
AML	PDI	54.60 ± 6.17^Ba^	59.33 ± 13.36^Ca^
	LCP	54.33 ± 3.64^Ba^	51.09 ± 9.85^Ca^
	NC	53.29 ± 4.35^Ba^	47.45 ± 3.46^Ca^

The use of different letters next to the ‘mean ± sd’ values indicate a statistically significant difference among means: uppercase for comparison in the same column and lowercase for comparison in the same line. Mixed ANOVA: time (*p* < 0.001); treatment (*p* = 0.696); resin (*p* < 0.001); time × treatment (*p* = 0.958); Time × resin (*p* < 0.001); resin × treatment (*p* < 0.001); time × resin × treatment (*p* < 0.001). Šidák correction (*p* ≤ 0.038).

**Table 4:** Mean values (±standard deviation) of roughness values (in µm) obtained before treatment and after treatment (PDI: photodynamic therapy; LCP: Liquid crystal precursor without curcumin; NC: negative control/artificial saliva) and organized by composite resin (TPH, Z350, and AML).

Composite Resin	Treatment	Evaluation Time	
		Before	After
TPH	PDI	0.095 ± 0.02^ABa^	0.093 ± 0.02^ABa^
	LCP	0.096 ± 0.01^Ba^	0.094 ± 0.01^Ba^
	NC	0.099 ± 0.02^Ba^	0.093 ± 0.01^Ba^
Z350	PDI	0.108 ± 0.03^BCa^	0.113 ± 0.04^BCa^
	LCP	0.098 ± 0.02^BCa^	0.116 ± 0.03^BCa^
	NC	0.101 ± 0.02^Ba^	0.104 ± 0.02^Ba^
AML	PDI	0.139 ± 0.09^BCa^	0.110 ± 0.07^BCa^
	LCP	0.162 ± 0.11^Ca^	0.171 ± 0.11^Ca^
	NC	0.078 ± 0.03^Aa^	0.078 ± 0.02^Aa^

The use of different letters next to the ‘mean ± sd’ values indicate a statistically significant difference among means: uppercase for comparison in the same column and lowercase for comparison in the same line. Mixed ANOVA: time (*p* = 0.991); treatment (*p* < 0.001); resin (*p* < 0.001); time × treatment (*p* = 0.369); time × resin (*p* = 0.457); resin × treatment (*p* < 0.001); time × resin × treatment (*p* = 0.703). Games Howell (*p* ≤ 0.034).

## Data Availability

Data available on request from the corresponding author.
